# Gut microbiomes of cyprinid fish exhibit host-species symbiosis along gut trait and diet

**DOI:** 10.3389/fmicb.2022.936601

**Published:** 2022-08-09

**Authors:** Yaqiu Liu, Xinhui Li, Yuefei Li, Jie Li, Shuli Zhu

**Affiliations:** ^1^Pearl River Fisheries Research Institute, Chinese Academy of Fishery Sciences, Guangzhou, China; ^2^Guangzhou Scientific Observing and Experimental Station of National Fisheries Resources and Environment, Guangzhou, China; ^3^Key Laboratory of Aquatic Animal Immune Technology of Guangdong Province, Guangzhou, China

**Keywords:** gut microbiome, Cyprinidae, coexistence, gut length, metabolism

## Abstract

Teleost omnivorous fish that coexist partially sharing resources are likely to modify their gut traits and microbiome as a feedback mechanism between ecological processes and evolution. However, we do not understand how the core gut microbiome supports the metabolic capacity of the host and regulates digestive functions in specialized omnivorous fish gut traits. Therefore, we evaluated the gut microbiome of eight omnivorous fish from a single family (i.e., Cyprinidae) in the current study. We examined the correlation between host phylogeny, diet composition, and intestinal morphological traits related to the intestinal microbiome. The results indicated that cyprinid fish with similar relative gut lengths had considerable gut microbiome similarity. Notably, the SL (short relative gut length) group, as zoobenthos and zooplankton specialists, was abundant in Proteobacteria and was less abundant in Firmicutes than in the ML (medium relative gut length) and LL (long relative gut length) groups. These fish could extract nutrients from aquatic plants and algae. Additionally, we found the relative abundance of *Clostridium* and *Romboutsia* to be positively correlated with host relative gut length but negatively correlated with the relative abundance of *Cetobacterium*, *Plesiomonas*, *Bacteroides*, and *Lactobacillus*, and host-relative gut length. We also show a positive linear relationship between host gut microbiome carbohydrate metabolism and relative gut length, while the amino acid and lipid metabolism of the gut microbiome was negatively correlated with host-relative gut length. In addition, omnivorous species competing for resources improve their ecological adaptability through the specialization of gut length, which is closely related to variation in the synergy of the gut microbiome. Above all, specialized gut microbiota and associated gut morphologies enable fish to variably tolerate resource fluctuation and improve the utilization efficiency of nutrient extraction from challenging food resources.

## Introduction

The co-evolution of animals and microbes within their guts facilitates their radiation into a wide variety of habitats (Muegge et al., 2011). In the long-term evolutionary process, hosts and microbes cooperate and interact, eventually forming an intimate symbiotic relationship (Suzuki, 2017). Over the past decade, our understanding of the diversity and functions of host-associated microbial communities has dramatically expanded (Ley et al., 2008; McFall-Ngai et al., 2013; Ye et al., 2014; Rennison et al., 2019). Notably, the adaptive capacity of an animal species is not determined solely by the host genome but must also include the vast genetic repertoire of the microbiome (Rosenberg and Zilber-Rosenberg, 2018). To gain a better understanding of the role of the gut microbiome in animal fitness, microbiome research is now considered a major area of research in ecology and evolution (Levin et al., 2021). Vertebrates consume a large array of food items, and their guts and microbiome reflect a complexity influenced by diet and genetics (Karasov and Douglas, 2013; Youngblut et al., 2019). Moreover, fish comprise the largest vertebrate group, with a wide spectrum of host habitats, physiology, and ecological strategies (Parris et al., 2016). Specifically, teleost fish are an invaluable repertoire of host species suitable for the study of factors shaping animal-associated microbiomes (Sylvain et al., 2020). Givens et al. (2015) found that some core shares OTUs in the guts of fish species. These may be important contributors to fish gut functions, such as digestion, nutrient absorption, and immune response. Several studies have shown that fish microbial effects can not only support the important role of microbes in promoting or enhancing fish adaptation but also potentially facilitate diversification (Huang et al., 2020; Liu et al., 2022). Moreover, numerous published studies have indicated that fish gut microbiota can be affected by factors, such as host diet, habitat, and genotypes (Li et al., 2020; Kang et al., 2022). However, these factors are often interrelated, and their effects on the fish gut microbiome tend to be complex and highly confounded.

Fish have specific gut traits that depend on their diet (Egerton et al., 2018). Nevertheless, it is not clear what gut trait specialization means for the gut microbiomes of fish. Fish gut length is one of the most important specialized gut traits in the aquatic ecosystem and can result in changes to the ecological interactions between species and their “fitness” in the community (Post and Palkovacs, 2009; Vasseur et al., 2011; Barabás and D’Andrea, 2016; Pastore et al., 2021). It is widely recognized that fish gut length can be affected by evolved differences in the diet as well as phylogenetic history (German and Horn, 2006; Zandonà et al., 2015). Longer guts are often observed in herbivorous fish, while shorter guts are often found in carnivorous fish (German and Horn, 2006). Nevertheless, omnivorous fish have a more diverse diet than herbivorous and carnivorous fish and the trophic niches overlapped. For instance, grass carp is considered an herbivorous fish. However, relevant findings show that the grass carp feeds primarily on Streptophyta and facultatively on Arthropoda, Rotifera, and Ascomycota, indicating that the grass carp is an omnivorous and only partially herbivorous fish (Zhang et al., 2021). Furthermore, the length of the gut determines the duration of food retention, whereas building and maintaining a long gut have high evolutionary and physiological costs. There is a trade-off between the benefits of nutrient acquisition and the costs of maintaining a long gut in a host (Ghilardi et al., 2021). A relevant study demonstrated that both evolutionary history and plasticity in diet quality can drive variation in fish gut length and the fish gut microbiome (Escalas et al., 2021). In recent years, an intensive study of wild fish gut microbes has mainly focused on the diversities and complexities of gut microbiota communities in wild fish species with different genotype and trophic levels (Liu et al., 2016; Escalas et al., 2021; Ofek et al., 2021). However, there is still limited information on relationship between the microbiomes of omnivorous fish hosts gut microbiome and their gut length.

Cyprinidae has more than 2,000 freshwater fish species, is widely distributed, and is one of the most diverse taxonomic groups in Cypriniformes. Cyprinid fish are composed of an abundance of omnivorous fish species that show incredible diversity, leading to resource specialization in various species, yet the gut length of these same species shows marked generality and plasticity, maintaining the ability to process a diversified diet. Thus, Cyprinidae is considered a good taxanomic group for exploring the gut microbiome of omnivorous fish in response to gut length specialization. However, the overall role of diet and anatomy in shaping the gut microbiome remains to be unfolded, especially within families characterized by ecological diversification. Given the general close relationship between the composition of the gut microbiome and digestion strategy, we hypothesized that there could be a possible link between gut length and gut microbiome in omnivorous fish, which have a diversified diet and a wide trophic niche. To assess our hypotheses, we utilized a high-throughput 16S ribosomal RNA (rRNA) sequencing to assess the gut microbiome of eight Cyprinidae species, namely, Grass carp (*Ctenopharyngodon idellus*, GC); Black Amur bream (*Megalobrama terminalis*, BA); Silver carp (*Hypophthalmichthys molitrix*, SC); Barbel chub (*Squaliobarbus curriculus*, BC); Bleeker’s yellow tail (*Xenocypris davidi*, XD); Topmouth culter (*Culter alburnus*, TC); Mud carp (*Cirrhinus molitorell*a, MC), and Common carp (*Cyprinus carpio*, CA) from a single location with the same cohabitated environment to gain insights into how host evolutionary history, diet, and gut anatomy are related to the gut microbiome. Additionally, studying the gut microbiomes of eight Cyprinidae species is greatly important for further understanding of symbiotic interactions between fish hosts and their microbes.

## Materials and methods

### Fish sample collection

**FIGURE 1:**
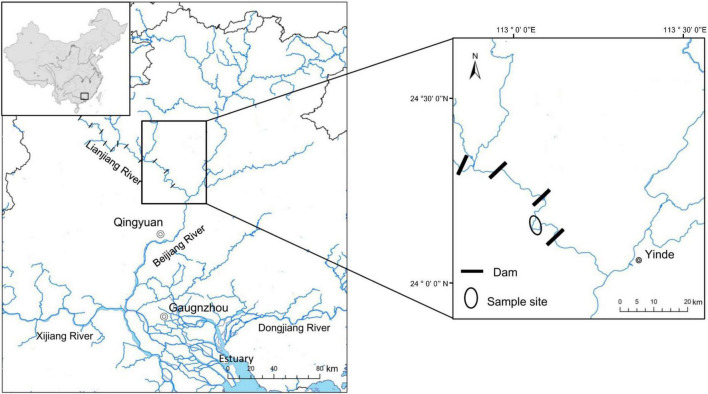
Location of the sample sites.

In this study, eight wild Cyprinidae fish species were collected from the Xiniu reservoir (one of the cascades in the Lianjiang River, owning 12 cascades. Fragmented fluvial habitats compress fish’s living space and lead to a relatively independent environment in different cascades, which is supposed to be an ideal minor natural ecosystem) in Lianjiang River, Guangdong Province, China in July 2021 (Figure 1). The Lianjiang River is the largest tributary of the Beijiang River, and has 12 cascades. We captured 80 fish using gillnets (10 specimens for each species). Basic environmental information of sample sites is provided in Supplementary Table 1. Body length (BL, to the nearest 1 mm) was measured from the length from the tip of the mandible to the base of the caudal fin; Body weight (BW was measured to the nearest 1 g), Gut morphology was characterized using gut length (mm), and relative gut length (gut length/body length) was measured. Relative gut length was used to reduce the effect of individual differences on gut length. Based on the discrepancy in relative gut length, eight cyprinid fish were divided into three groups, the SL (short: relative gut length < 2); ML (medium: 2 ≤ relative gut length < 4); and LL (long: relative gut length ≥ 4) groups. In order to investigate the fish gut microbiota, three or four fish were randomly selected for sequencing from eight species samples. Prior to dissection, fish were euthanized with an overdose of MS 222 (3-aminobenzoic acid ethyl ester methanesulfonate, Sigma, Germany). All procedures for the handling of wild freshwater fish species were approved by the institutional animal care. To prevent contamination from the skin surface, and eliminate transient bacteria, the whole intestinal tract of an individual fish sample was dissected with sterile instruments and quickly washed in 75% ethanol and sterile water. The obtained gut contents that were utilized for deoxyribonucleic acid (DNA) extraction were immediately put into liquid nitrogen and then transferred to an ultra-low temperature freezer and stored at –80°C until use.

### Deoxyribonucleic acid extraction and amplification

Approximately, 0.2 g of each sample was extracted using a QIAamp DNA Stool Mini Kit (Qiagen, Valencia, United States). All DNA extracts were stored at –80°C until use. The V3-V4 hypervariable region of the bacterial 16S rRNA genes was amplified using the specific primer pairs 341F (5′-CCTAYGGGRBGCASCAG-3′) and 806R (5′- GGACTACNNGGGTATCTAAT-3′) by an ABI GeneAmp^®^ 9700 PCR thermocycler (ABI, CA, United States). Total DNA from the gut of different fish samples was sent to Novogene Bioinformatics Technology, Co., Ltd., Beijing, China for further sequencing analysis.

### High-throughput sequencing analysis

Sequencing libraries were created using the Ion Plus Fragment Library Kit 48 rxns (Thermo Scientific, United States) according to the manufacturer’s instructions. The library quality was assessed on a Qubit@ 2.0 Fluorometer (Thermo Scientific, United States). After that, the library was sequenced, and single-end reads were generated. Quality filtering of the raw reads was performed under specific filtering conditions to obtain high-quality clean reads according to the cut-adapt quality-controlled process (Martin, 2011). The tags were compared with the reference database using the UCHIME algorithm to detect chimera sequences (Edgar et al., 2011; Haas et al., 2011). Sequence analysis was performed using UPARSE 7.1 software (Edgar, 2013). Sequences with equal or greater than 97% similarity were assigned to the same operational taxonomic unit (OTU). The most abundant sequence for each OTU was selected as a representative sequence. To minimize the effects of sequencing depth on alpha and beta diversity measure, the number of 16S rRNA gene sequences from each sample was rarefied to 20,000, which still yielded an average Good’s coverage of 99.09%. To predict the microbial function of the bacterial communities on the gut contents of different samples, PICRUSt (Phylogenetic Investigation of Communities by Reconstruction of Unobserved States) was utilized to analyze the Kyoto Encyclopedia of Genes and Genomes (KEGG) pathways at Levels 2 and 3 (Douglas et al., 2020).

### Phylogenetic analysis of fish

Phylogenetic analysis of eight Cyprinid species (grass carp, black Amur bream, silver carp, barbel chub, bleeker’s yellowtail, topmouth culter, mud carp, and common carp) based on cytochrome c subunit I (CO1) gene was done using the MEGA program (version 7.0) (Kumar et al., 2016). The corresponding gene sequences were downloaded from GenBank (accession numbers in Supplementary Table 2). The concatenated sequences of the mitochondrial genes were aligned using ClustalW in MEGA (pairwise and multiple alignment parameters). The phylogenetic tree was constructed based on the aligned DNA sequences by the neighbor-joining method using the Kimura 2-parameter model in MEGA, and then pairwise genetic distance among eight cyprinid species was calculated using MEGA (Kumar et al., 2016).

### Statistical analysis

The unweighted pair-group method with arithmetic means (UPGMA) clustering was performed to interpret the Bray-Curtis distance matrix using average linkage, which is a hierarchical clustering method. It was performed using QIIME software (Version 1.9.1). Then, we used Mantel tests implemented in the *R* software package to assess the relationships between host phylogenetic and microbiome dissimilarity matrixes (Goslee and Urban, 2007). Based on the OTU information, alpha-diversity indices, including the Shannon index and ACE richness index, were calculated using QIIME (version 1.9.1). The shared and unique OTUs of different groups were also represented by a Scale-Venn diagram. A one-way ANOVA test was used in SPSS Statistics 28.0 to evaluate if the differences in alpha diversity between groups were significant. A *p*-value below0.05 was used to determine statistical significance. Significance tests of the bacterial community composition with analysis of similarities (ANOSIM) were computed with 999 permutations using the VEGAN package and carried out on the significance of defined categories based on Bray-Curtis distances using the OTU table (Clarke, 1993). Power analysis for the ANOVA-based and permutation-based ANOSIM test was used to estimate sample size (Cohen, 1977; Wang and Zhang, 2021; Supplementary Figures 1, 2). Adonis analysis (on parametric MANOVA) based on Bray-Curtis distances was used to explain the different grouping factors for differences of samples, and the statistical significance of division was analyzed for significance using a permutation test (QIIME version 1.9.1). Non-metric multidimensional scaling (NMDS) was used to analyze the differences in the bacterial community composition of different groups across orders and phyla based on the Bray-Curtis distance matrix using the VEGAN package (Lozupone and Knight, 2005). A principal coordinate analysis (PCoA) was conducted to explain the difference between the KOs of different groups of relative gut length by using the WGCNA package, stats, and ggplot2. Here, we used the *R* implementation of the procedure (version 3.1.14). For correlation analysis, the relationships between the relative gut length and alpha diversity or the relative abundance of core genera relative abundance and digestion-related bacterial gene functions were investigated separately, and the *p*-values under0.05 were considered indicative of significant pairwise relationships.

## Results

### Relationships between host and core microbiome dissimilarity

**FIGURE 2:**
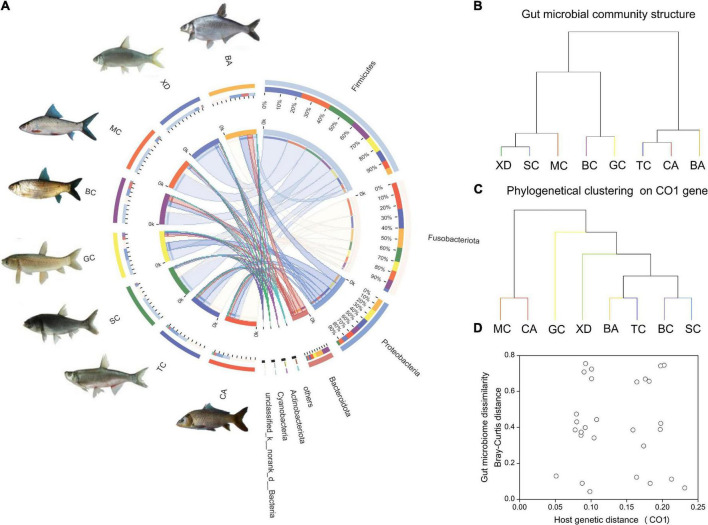
An overview of the data. **(A)** A circos chart indicating microbial communities in fish gut samples at the phylum level. The inner circular diagram shows the relative abundance of different phyla in different cyprinid fish gut samples. Only those with mean relative abundance more than 1% for phylum are shown. Sequences that could not be assigned at the phylum level were marked as “Unclassified”; **(B)** hierarchical clustering of gut microbiome of the different cyprinid fish based on Bray-Curtis distance; **(C)** phylogenetical clustering based on host genetic distance (the CO1 gene); **(D)** relationship between fish gut microbiome dissimilarity based on Bray-Curtis distance and host genetic distance (the CO1 gene). BA, Black Amur bream; BC, Barbel chub; CA, Common carp; GC, Grass carp; MC, Mud carp; SC, Silver carp; TC, Topmouth culter; XD, Bleeker’s yellow tail.

The gut bacteria of the eight different cyprinid fish species showed remarkable differences. At the phylum level, the gut bacteria of TC, CA, and BA were dominated by Fusobacteriota (41.7–57.3%); those of BC and GC were characterized by Firmicutes (35.3–43.9%) and Proteobacteria (19.–26.8%), and those of XD, SC, and MC were dominated by Firmicutes (63.8–73.6%) (Figure 2A). It is evident from the results that, overall, with the same diet type and gut trait, the community composition was similar (Table 1 and Figure 2B). We generated an UPGMA tree based on 16S rDNA gene sequences from the gut microbial communities (Figure 2B). Phylogenetic analysis of eight Cyprinid species based on the cytochrome c subunit I (CO1) gene was indicated in Figure 2C. As shown in Figure 2D, the CO1 genetic distance between fish species was not correlated with their microbiome dissimilarity (*p* > 0.05).

**TABLE 1:** An overview of fish samples, including biological information and dietary composition.

Host	*n*	Body weight	Body length	Gut length	Gut length/body length ratio	Relative gut length level	Dietary composition		
							
							Dominant food item	Facultative food item	References
TC	10	330 ± 10.54	249 ± 15.41	214 ± 22.98	0.86	SL	Zooplankton (crustaceans); zoobenthos (insects); small fish	Other aquatic plant (vascular plant).	Zheng, 1989; Zhou and Zhang, 2005; Froese and Pauly, 2022
CA	10	473 ± 21.65	248 ± 16.98	345 ± 31.25	1.39	SL	Zoobenthos (aquatic insects; mollusks); Zooplankton (cladocerans; crustaceans)	Phytoplankton (algae); other aquatic plant (terrestrial plants).	Zheng, 1989; Lu, 1990; Froese and Pauly, 2022
BC	10	323 ± 23.61	269 ± 20.41	557 ± 48.97	2.08	ML	Zooplankton (fish eggs/larvae; planktonic crustaceans); Phytoplankton (algae)	Zoobenthos (aquatic insects).	Zheng, 1989; Lu, 1990
GC	10	319 ± 20.48	253 ± 16.47	552 ± 38.95	2.18	ML	Other aquatic plant (terrestrial plants); phytoplankton (algae)	Zooplankton (invertebrates); zoobenthos (aquatic insects).	Lu, 1990; Zhang et al., 2021; Froese and Pauly, 2022
BA	10	316 ± 18.97	229 ± 15.87	657 ± 50.14	2.87	ML	Zooplankton (crustaceans); zoobenthos (mollusk)	other aquatic plant (terrestrial plants); Phytoplankton (algae).	Lu, 1990; Xia et al., 2017
XD	10	271 ± 23.41	199 ± 20.14	1147 ± 106.72	5.77	LL	Phytoplankton (algae); Other aquatic plant (terrestrial plants)	Zooplankton (invertebrates); zoobenthos (aquatic insects).	Zheng, 1989; Lu, 1990; Froese and Pauly, 2022
SC	10	408 ± 28.64	276 ± 22.98	1779 ± 123.12	6.44	LL	Phytoplankton (algae); zooplankton (invertebrates)	Zoobenthos (aquatic insects).	Zheng, 1989; Lu, 1990; Froese and Pauly, 2022
MC	10	339 ± 19.52	244 ± 17.54	2432 ± 198.64	9.98	LL	Phytoplankton (algae); zooplankton (invertebrates)	Zoobenthos (invertebrates); other aquatic plant (terrestrial plants).	Zheng, 1989; Lu, 1990; Froese and Pauly, 2022

Relative gut length groups (SL: gut length/body length < 2; ML: 2 ≤ gut length/body length < 4; LL: gut length/body length ≥ 4). BA, Black Amur bream; BC, Barbel chub; CA, Common carp; GC, Grass carp; MC, Mud carp; SC, Silver carp; TC, Topmouth culter; XD, Bleeker’s yellow tail.

### Association between cyprinid gut microbiome and specialized gut traits

**FIGURE 3:**
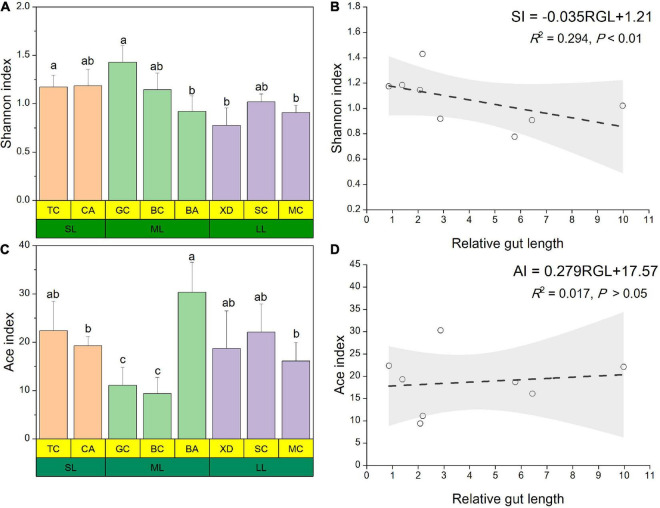
Alpha diversity results of the gut microbial community pertaining for the eight different fish species at three relative gut length levels. The Shannon **(A)** and Ace **(C)** index of gut microbiota composition from the eight different fish species at three relative gut length levels. Samples marked different lowercase letters indicate significant differences (a > b > c; *p* < 0.05) among different fish species. Linear regression of the Shannon **(B)** and Ace **(D)** index among eight cyprinid fish species vs. mean of their relative gut length with 95% confidence interval. BA, Black Amur bream; BC, Barbel chub; CA, Common carp; GC, Grass carp; MC, Mud carp; SC, Silver carp; TC, Topmouth culter; XD, Bleeker’s yellow tail; RGL, Relative gut length; SI, Shannon index; AI, Ace index.

**FIGURE 4:**
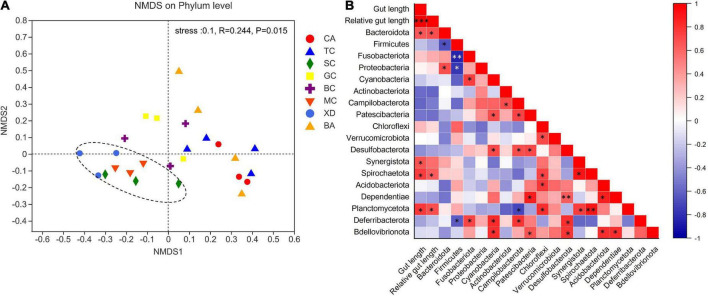
An overview of the data. **(A)** NMDS (non-metric multidimensional scaling) based on bray curtis distance matrix, demonstrating different fish samples. Significance tests of the bacterial community composition with analysis of similarities (ANOSIMm), indicating the significance of defined species based on Bray-Curtis distance. The individual samples are color-coordinated according to the different species. **(B)** Pearson correlation analysis between relative gut length and dominant phylum in the eight fish species *p < 0.05; **p < 0.01; ***p < 0.001. BA, Black Amur bream; BC, Barbel chub; CA, Common carp; GC, Grass carp; MC, Mud carp; SC, Silver carp; TC, Topmouth culter; XD, Bleeker’s yellow tail.

The microbial complexity in eight fish species was estimated on the basis of alpha-diversity (Shannon and Ace indices), and it showed distinct differences (Figures 3A,C). The Shannon index of the SL (short-relative gut length) and ML (medium-relative gut length) groups was larger than that of the LL (long-relative gut length) group, whereas there were no significant differences among the three groups. It should be noted that alpha-diversity indices exhibit obvious differences even in the similar relative gut length level. We observed that there was a linear relationship between Shannon index and relative gut length, while no significant correlation was found between Ace index and relative gut length (Figures 3B,D). Furthermore, similarities of the microbial community composition between fish samples were compared by NMDS based on Bray-Curtis distance (Figure 4A). The XD, SC, and MC samples formed a cluster and distinctly separated from the cluster of CA and TC samples, while others were located in the middle of them. In addition, we established significant differences of gut microbiome among different fish species (ANOSIM analysis: *R* = 0.244, *p* = 0.015; Adonis analysis: *R*^2^ = 0.495, *p* = 0.006). Pearson correlation analysis between gut trait and dominant phylum in cyprinid fish species showed that the gut length and relative gut length were significantly correlated with the relative abundance of Bacteroidota and Spirochaetota (Figure 4B). Additionally, there was a negative relationship between the Firmicutes and Proteobacteria and Fusobacteriota, while Proteobacteria showed a positive relationship with Bacteroidota in all the fish samples.

### Gut microbiome differentiation along the relative gut length

**FIGURE 5:**
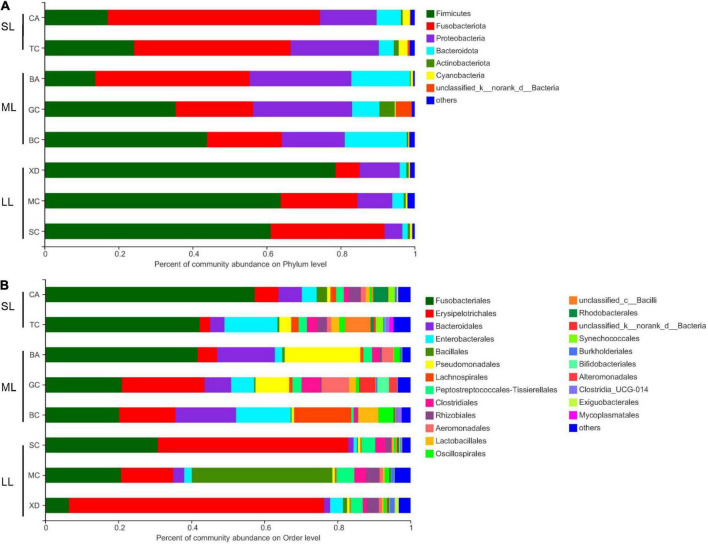
Comparison of the bacterial community in the different relative gut length groups. Dominant gut microbiota composition in the different groups at the phylum **(A)** and order **(B**) level; each bar represents average relative abundance of each bacterial taxon within a group at the phylum and order level. BA, Black Amur bream; BC, Barbel chub; CA, Common carp; GC, Grass carp; MC, Mud carp; SC, Silver carp; TC, Topmouth culter; XD, Bleeker’s yellow tail.

**FIGURE 6:**
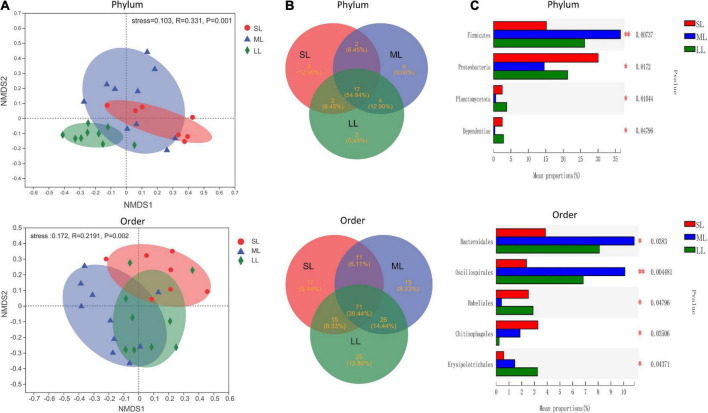
An overview of the data. **(A)** NMDS based on the bray curtis distance matrix demonstrating different fish samples at the phylum and order level. The individual sample is color-coordinated according to the different gut length groups; significance tests of the bacterial community composition with analysis of similarities (ANOSIM), indicating the significance of groups based on Bray-Curtis distances. **(B)** The Venn diagram illustrating the shared and unique phylum and orders in the different gut length groups. **(C)** One-way ANOVA analysis demonstrating significant differences of gut bacterial phyla and order in the different gut length groups. Samples marked by an asterisk indicate significant differences (*p < 0.05; ^**^p < 0.01) among relative gut length groups.

**FIGURE 7:**
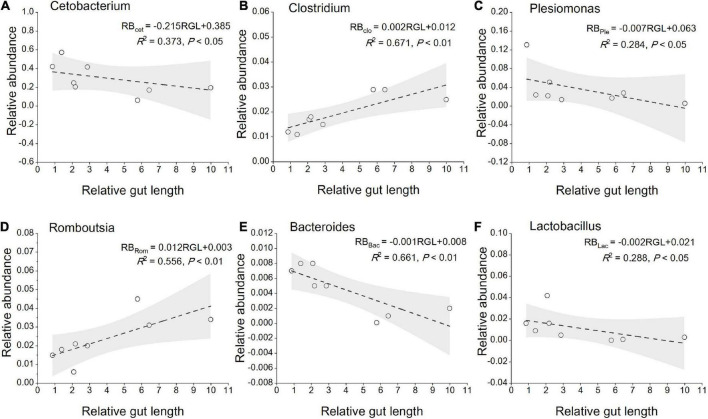
Linear regression of relative abundance of gut core microbiome (the genera level) among eight cyprinid fish species vs. mean of their relative gut length, with 95% confidence interval. **(A)** Cetobacterium; **(B)** clostridium; **(C)** plesiomonas; **(D)** romboutsia; **(E)** bacteroides; **(F)** lactobacillus. RGL, Relative gut length; RB, Relative abundance; Cet, Cetobacterium; Clo, Clostridium; Ple, Plesiomonas; Rom, Romboutsia; Bac, Bacteroides; Lac, Lactobacillus.

The most abundant taxa of bacteria in each relative gut length group were observed at the phylum and order levels (Figures 5A,B). Firmicutes were the most abundant in the XD, MC, and SC groups, whereas the most abundant phylum of the CA and TC groups was the Fusobacteriota (Figure 5A). According to Figure 5B, Fusobacteriota was observed as the dominant order of the CA and TC groups, while the dominant orders of the SC and XD groups were Lachnospirales. Moreover, we compared similarities of the microbial community composition among the three groups by NMDS based on Bray-Curtis distance at the phylum and order levels (Figure 6A). At the phylum level, SL samples formed a cluster and distinctly separated from the cluster of LL samples, while ML samples were located in the middle of them (ANOSIM analysis: *R* = 0.331, *p* = 0.001; Adonis analysis: *R*^2^ = 0.332, *p* = 0.001). Nevertheless, SL samples separated from the cluster of the LL and ML samples, which were close (Anosim analysis: *R* = 0.219, *p* = 0.002; Adnois analysis: *R*^2^ = 0.251, *p* = 0.001). Furthermore, the Venn diagram revealed that the three groups shared 17 phyla, whereas the ML group shared the fewest unique phyla. The number of common orders presented in all groups was 71, and unique orders for each group varied from 15 to 25 (Figure 6B). The ML group shared more orders with the LL group than with the SL group. A one-way ANOVA analysis of the dominant microbiome among the three groups showed significant differences in Firmicutes, Proteobacteria, Planctomycetota, and Dependentiae (*p* < 0.05) (Figure 6C and Table 2). At the order level, the relative abundance of Chitinophagales in the SL group was the highest, whereas the relative abundance of Erysipelotrichales, Bacteroidales, and Osillospirales was the lowest among the three groups (*p* < 0.05). The relative abundance of Babeliales in the ML group was much lower than that of the LL groups (*p* < 0.05) (Figure 6C and Table 2). In addition, it is evident from the result that there was a positive linear relationship between the relative abundance of *Clostridium* and *Romboutsia* and host relative gut length, whereas a negative correlation between the relative abundance of *Cetobacterium*, *Plesiomonas*, *Bacteroides*, and *Lactobacillus* and the host relative gut length was observed (Figure 7).

**TABLE 2:** One-way ANOVA analysis of differences of gut bacterial phyla and order in the different relative gut length groups.

	SL vs. ML	ML vs. LL	SL vs. LL
	*P*-value	*P*-value	*P*-value
**Phylum**			
p__Firmicutes	0.157	0.001**	0.001**
p__Proteobacteria	0.807	0.094	0.004**
p__Planctomycetota	0.107	0.013*	0.085
p__Dependentiae	0.107	0.010*	0.459
**Order**			
o__Bacteroidales	0.048*	0.130	0.042*
o__Oscillospirales	0.039*	0.137	0.037*
o__Babeliales	0.107	0.010*	0.244
o__Chitinophagales	0.046*	0.870	0.044*
o__Erysipelotrichales	0.047*	0.025*	0.008**

*Means significant difference between two populations (p < 0.05). **Means very significant difference between two populations (p < 0.01).

### Predicted gut microbiome digestion-related function using phylogenetic investigation of communities by reconstruction of unobserved states

**FIGURE 8:**
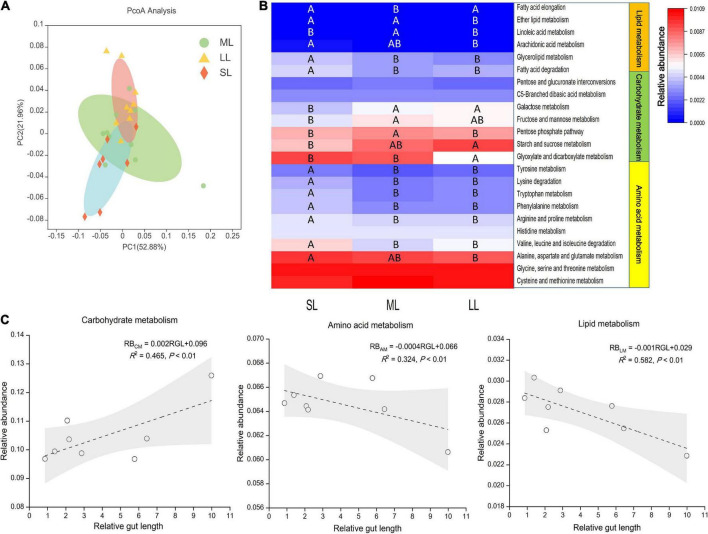
KEGG categories derived from the 16S rRNA sequences of the fish gut microbiomes by PICRUSt. **(A)** PCoA of the binary Jaccard dissimilarity of the functional profiles (Level 3). **(B)** The Heatmap presenting the relative abundance of digestion-related bacterial gene functions among the three gut length groups. Samples marked different capital letters indicate significant differences (A > B > C; *p* < 0.05) among relative gut length groups. **(C)** Linear regression of relative abundance of digestion-related bacterial gene functions among eight Cyprinidae fish species vs. mean of their relative gut length, with 95% confidence interval.

Here, we aimed to investigate a possible link between gut traits and gut microbiome digestion-related functional profiles by utilizing the PICRUSt pipeline. KEGG ortholog groups (KOs) were predicted by PICRUSt. PCoA based on KOs (Level 3) revealed that there was an obvious distinct separation of functional gene distribution between the SL and LL groups (Figure 8A). We identified 23 pathways related to digestion, including carbohydrate, amino acid, and lipid metabolism, of which 19 pathways showed significant differences in relative abundance among the three groups (Figure 8B). Some of the amino acid metabolism pathways (i.e., alanine, aspartate, glutamate, valine, leucine, and proline pathways) were highly abundant in the SL group. For some pathways related to carbohydrate metabolism, starch and sucrose metabolism was more enriched in the LL group than in the SL group, while fructose, mannose, and pentose metabolism was more enriched in the ML group. Some pathways related to lipid metabolism (i.e., fatty acid degradation and glycerolipid and ether fatty acid metabolism) were more enriched in the SL group than in the ML and LL groups. There was a positive linear relationship between gut microbiome carbohydrate metabolism and relative gut length, whereas a negative correlation between gut microbiome amino acid, lipid metabolism, and relative gut length was observed (Figure 8C).

## Discussion

In this study, the dominant microbiome groups in eight cyprinid species were Firmicutes, Fusobacteriota, Proteobacteria, and Bacteroidetes. These four phyla represented more than 80% of the sequences, which is consistent with a previous research, indicating the commonality of many fish gut communities (Ghanbari et al., 2015). Moreover, the relative abundance of the same phylum in the different species showed an obvious divergence (Figure 2A). Several studies have established that fish gut microbial community compositions are distinct among different species, even among those co-habiting in the same environment (Eichmiller et al., 2016; Baldo et al., 2017). Differentiation of the fish gut microbiome can frequently be predicted by host variable exogenous diet, environmental conditions, and phylogeny (Fishelson et al., 1985; Su et al., 2020; Liu et al., 2021). In this study, we preliminarily investigated the relationship between cyprinid fish hosts CO1 genetic distance and gut microbiome dissimilarity. No obvious correlation signal was observed, which is consistent with the study of Escalas et al. (2021). Relevant research has shown that host genetic relatedness based on variation in the CO1 gene showed no significant association between similarity in the composition of the gut microbial community and host phylogenetic distance by investigating gut microbiota of 85 different fish species (Kim et al., 2021). The fish gut microbiome is affected by multiple complex factors, especially in the natural aquatic ecosystem. Levin et al. (2021) also found that the composition, diversity, and function of gut microbes in wild animals are closely related to host species, habitat, dietary habits, circadian rhythms, and social structure by analyzing the gut microbiota of over 180 species in the wild. Specifically, the host dietary strategy was also considered as one of the most important factors to influence the gut microbiome (Ley et al., 2008; Youngblut et al., 2019). Regardless of the phylogenetic distance among hosts, the dissimilarities of gut microbial communities between fish taxa were randomly distributed (Kim et al., 2021). Therefore, we speculated that it is hard to discern whether host genetic factors have a direct effect on the physiological control of the gut microbiome.

For alpha-diversity analysis, we found that the Shannon index of fish samples was negatively associated with their relative gut length (Figure 3B). These correlations were relatively low but significant. Relevant research demonstrated that gut morphology and diet were the strongest determinants of the gut microbiome in Sparidae (Escalas et al., 2021). Fish gut length and relative gut length were found to be conserved in host phylogeny and were regarded as an important specialized trait for exogenous resource utilization (Greene et al., 2020). Here, we found a clear separation between XD, SC, and MC samples and CA and TC samples (Figure 4A), indicating that omnivorous fish gut microbiome had a close relationship with their relative gut length. Gut length is central to one of the most important organismal processes, namely, the digestion of prey sourced from the environment, and, as such, it is likely to close links to functional roles (Ghilardi et al., 2021). Furthermore, gut length has already been proved to be negatively correlated with the trophic level in fish (Elliott and Bellwood, 2003). In the current study, we found fish gut length and relative gut length to be significantly correlated with the relative abundance of Bacteroidota. In general, consumers with longer guts can acquire more energy and nutrients from low nutritional food (i.e., aquatic plants and algae). Some previous studies have shown that Bacteroidota can assist the host by utilizing exogenous nutrition by the efficient degradation of polysaccharides (i.e., cellulose), which the host alone would not be able to degrade efficiently (Glenwright et al., 2017; Greene et al., 2020).

At the beta-diversity level, the gut microbiome of the SL and LL groups had an obvious differentiation, potentially indicating differences in the competition and utilization of exogenous nutrition (Roggenbuck et al., 2014; Greene et al., 2020). The SL group includes zoobenthos and zooplankton specialists, and their gut microbiota comprised more abundant Proteobacteria and less-abundant Firmicutes than the ML and LL groups (Figure 6C). It has been found that the function of these Proteobacteria is typically associated with shorter gastrointestinal systems, and only rarely with plant fiber (Dill-McFarland et al., 2016; Greene et al., 2019). Additionally, related research indicates that Proteobacteria are dominant in carnivorous fish (Kim et al., 2007; Merrifield et al., 2009; Liu et al., 2016). Firmicutes were more abundant than Proteobacteria in hosts with herbivorous diet (Gharechahi et al., 2021). In this study, we identified a dramatically different relative abundance of Oscillospirales and Chitinophagales among the three groups (Figure 6C). Further research has proved that Oscillospirales can help the host to ferment complex plant carbohydrates (Konikoff and Gophna, 2016; Gharechahi et al., 2021). As shown by previous research, some species in the order Chitinophagales can degrade chitin and organic matter (Hou et al., 2021). We found a negative correlation between gut core microbiome (*Cetobacterium*, *Bacteroides*, *Lactobacillus*) and relative gut length. According to previous research, *Cetobacterium* in the gut of fish host is closely linked to host protein digestion (Larsen et al., 2014; Liu et al., 2016). One vital gut microflora, *Lactobacillus*, can effectively help some animal hosts degrade polyunsaturated fatty acids (Falcinelli et al., 2015). Moreover, *Bacteroides* plays an important role in fish host glucose and lipid metabolism (Bjursell et al., 2011). The relative abundance of *Clostridium* increased with relative gut length, indicating potential enhancement of fish host carbohydrate metabolism, such as in the degradation of plant polysaccharides (Larsen et al., 2014).

Further prediction analysis of microbial metabolic function indicated that the LL group had a weaker amino acid and lipid metabolism, and stronger carbohydrate metabolism than the SL group. Our result also provides evidence of a positive linear relationship between gut microbiome carbohydrate metabolism and relative gut length, and a negative correlation between gut microbiome amino acid lipid metabolism, and relative gut length (Figure 8C). The result is consistent with the idea that longer gut length is associated with lower nutrient levels (Olsson et al., 2007; Zandonà et al., 2015; Ghilardi et al., 2021). Related research reveals that intestinal microbes play an important role in host nutrient metabolism, reflecting the host’s utilization of an exogenous diet (Huang et al., 2020; Zhu et al., 2021). On the whole, we found discrepancy in the gut microbiota of multiple trophic niches overlapping species, which could potentially reflect exogenous resources utilized efficiency of these species in similar habitat and environments (De León et al., 2014).

Cyprinid fish species may coexist because they have differentiated resource use and thus do not significantly interact, or they can coexist with partially overlapping resources use if they are nearly equivalent in their average competitive abilities (Yang and Hui, 2020). Dietary partitioning is one axis by which sympatric species avoid competition, and is typically documented by cataloging diets to show a minimal overlap (German and Horn, 2006; Ghilardi et al., 2021; Herrera et al., 2022). Omnivorous species that compete with each other for resources improve their ecological adaptability through gut trait regulation. A relevant researcher has suggested that morphological variation can promote bat dietary niche evolution (Chang et al., 2019). Gut length and associated gut microbiota specialization are closely related to changes in host dietary niche differentiation as compensation for species coexistence (Saavedra et al., 2017; Yang and Hui, 2020). In addition, the gut microbiome that inhabits fish guts is metabolically versatile, and, *via* its role in digesting substrates otherwise unavailable to hosts, they can help the host to expand their dietary options (Greene et al., 2020). Specifically, specialized gut microbiota and associated gut morphology enable fish in the LL group to variably tolerate resource fluctuation and support nutrient extraction from challenging resources (e.g., metabolizing plant secondary compounds or recalcitrant fibers), perhaps ultimately facilitating host species diversity and specialized feeding ecologies. Additionally, intestinal microbes in different fish species showed significant different utilization efficiency for exogenous food resources, and this may be one of the potential factors in improving host ecological adaptability in aquatic ecosystems. Nevertheless, how the underlying effects of the gut microbiome affect multi-fish species coexistence and trophic differentiation is still unclear. Therefore, the functional role of the colonization of symbiosis microbiome in fish host ecological adaptability needs further evaluation.

## Data availability statement

The datasets presented in this study can be found in online repositories. The names of the repository/repositories and accession number(s) can be found in the article/Supplementary material.

## Ethics statement

The animal study was reviewed and approved by the methods involving animals in this study were conducted in accordance with the Laboratory Animal Management Principles of China. All experimental protocols were approved by the Ethics Committee of the Pearl River Fisheries Research Institute, Chinese Academy of Fishery Sciences.

## Author contributions

YQL: data collection, conceptualization, data curation and analysis, and writing—original draft. XL: writing—review and editing. JL: funding acquisition and supervision. YFL: formal analysis. SZ: data collection. All authors contributed to the article and approved the submitted version.
