# Supplementary material for: Gut microbiomes of cyprinid fish exhibit host-species symbiosis along gut trait and diet
Source: Front Microbiol. 2022 Aug 9;13:936601. doi: 10.3389/fmicb.2022.936601 (PMC9396210; doi:10.3389/fmicb.2022.936601)
Supplement: Supplementary file 1 [file Data_Sheet_1.docx]

**SUPPLEMENTAL MATERIALS**


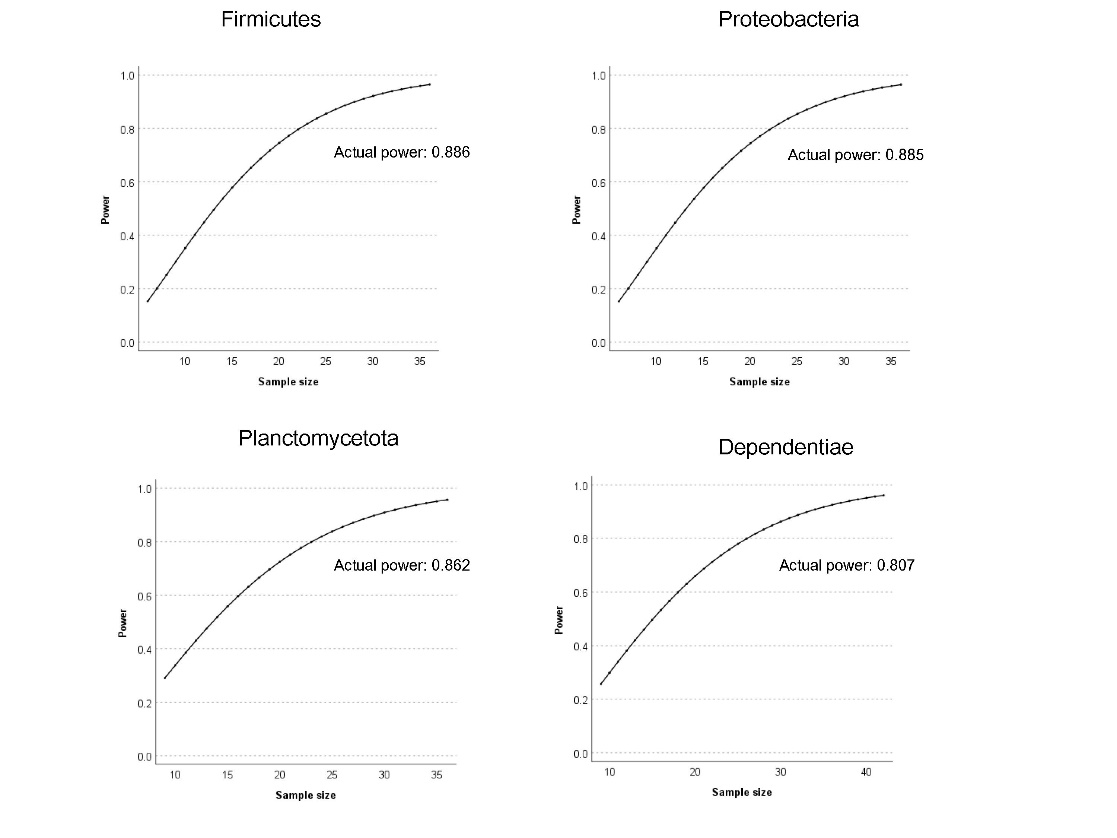


a

a


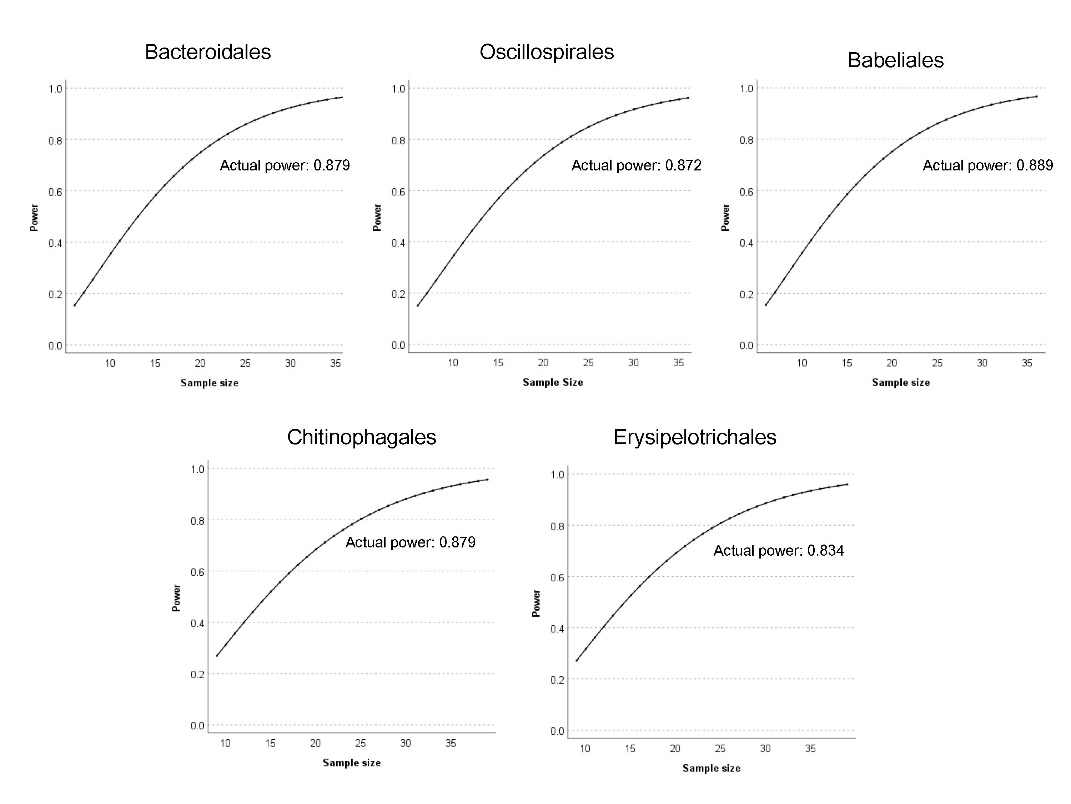


b

Supplementary Fig. S1 Power analysis estimating sample size for the ANOVA-based test on significant relative abundance of different groups in phylum (a) and order (b) level


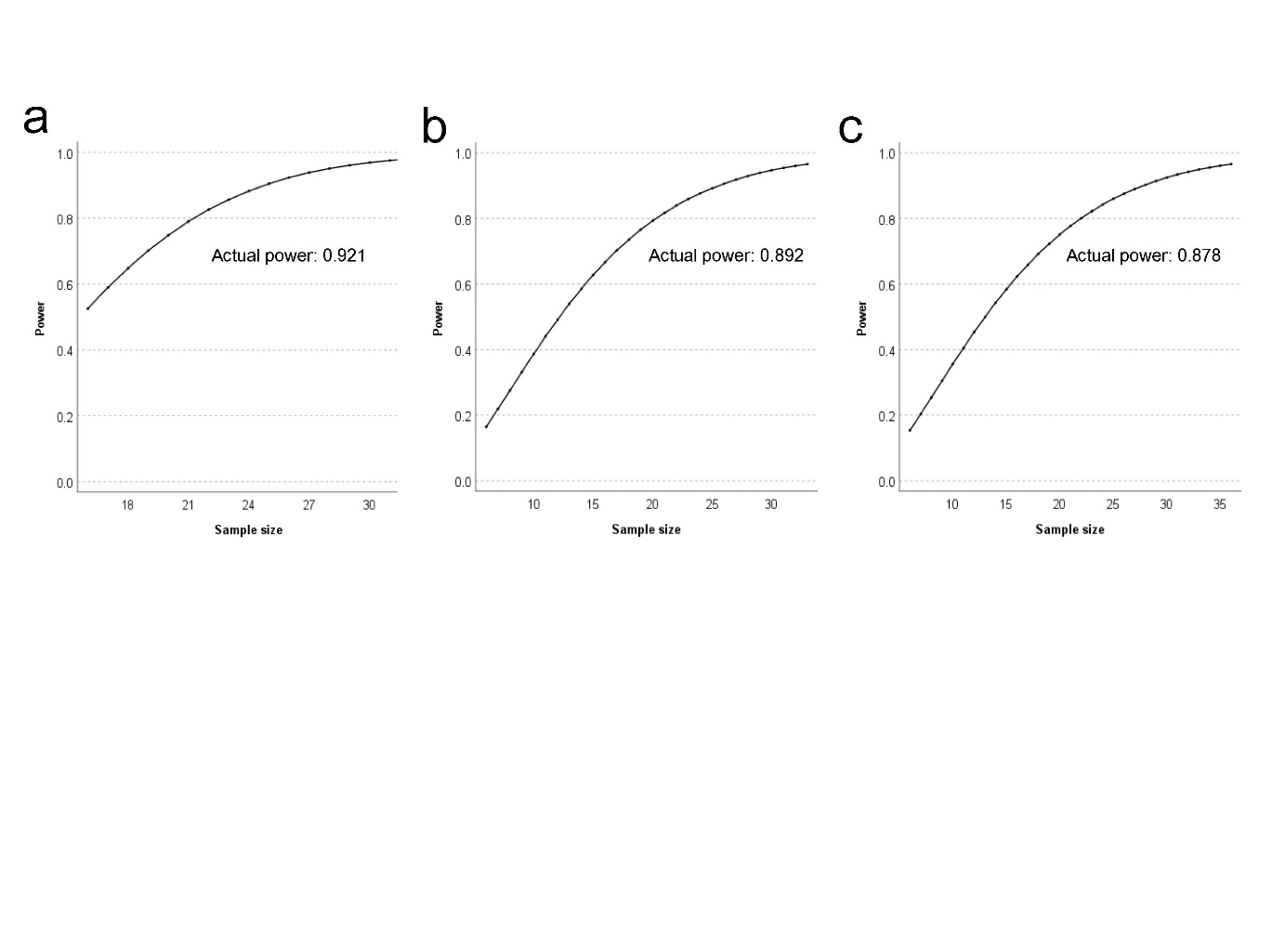


Supplementary Fig. S2 Power analysis estimating sample size for the permutation-based ANOSIM test of the bacterial community composition in different species (a), in the different gut length groups (b. phylum and c .orders).

Supplementary Table S1 Environmental factors of sample sites in the Lianjiang River

| Environmental factors | Range | [Instrument](javascript:;) and method |
| --- | --- | --- |
| Water temperatures | 24.2 ~ 24.6℃ | HQ30, Hach Company, Loveland, CO, USA |
| Dissolved Oxygen | 7.6 ~ 8.0 mg/L | HQ30, Hach Company, Loveland, CO, USA |
| pH | 7.1~7.2 | HQ30, Hach Company, Loveland, CO, USA |
| Flow rate | 0.19 ~ 0.23m/s | Data come from Gaodao hydrology station in the Lianjiang River |
| Discharge | 250 ~ 300 m^3^/s | Data come from Gaodao hydrology station in the Lianjiang River |
| Diaphaneity | 0.35 ~ 0.45m | Transparency was measured using a white Secchi Disc with a diameter of 30 cm. |
| Sediment concentration | 0.038 ~ 0.04 kg/m^2^ | Data come from Gaodao hydrology station in the Lianjiang River |

Supplementary Table S2 Accession numbers of different fish species downloaded from GenBank

| Host | Latin name | Accession numbers |
| --- | --- | --- |
| TC | *Culter alburnus* | MF122235.1 |
| CA | *Cyprinus carpio* | JX983284.1 |
| BC | *Squaliobarbus curriculus* | MW379796.1 |
| GC | *Ctenopharyngodon idella* | MT884525.1 |
| BA | *Megalobrama terminalis* | MT884624.1 |
| XD | *Xenocypris davidi* | MT884829.1 |
| SC | *Hypophthalmichthys molitrix* | JX983319.1 |
| MC | *Cirrhinus molitorella* | MT884504.1 |
